# The Male Reproductive Toxicity Caused by 2-Naphthylamine Was Related to Testicular Immunity Disorders

**DOI:** 10.3390/toxics12050342

**Published:** 2024-05-07

**Authors:** Pengyuan Dai, Mengqian Ding, Jingyan Yu, Yuan Gao, Miaomiao Wang, Jie Ling, Shijue Dong, Xiaoning Zhang, Xuhui Zeng, Xiaoli Sun

**Affiliations:** 1 Institute of Reproductive Medicine, Medical School, Nantong University, Nantong 226019, China; pengyuandai@ntu.edu.cn (P.D.); 2231310058@stmail.ntu.edu.cn (M.D.); 2231310053@stmail.ntu.edu.cn (J.Y.); wmm@stmail.ntu.edu.cn (M.W.); lingj@stmail.ntu.edu.cn (J.L.); 2113310047@stmail.ntu.edu.cn (S.D.); zhangxn@ntu.edu.cn (X.Z.); 2 Experimental Animal Center, Nantong University, Nantong 226001, China; dayuanzi@ntu.edu.cn; 3 Center for Reproductive Medicine, The Affiliated Hospital of Nantong University, Nantong University, Nantong 226001, China

**Keywords:** 2-naphthylamine, testis, sperm motility, malformation, lipid metabolism, immunity

## Abstract

2-naphthylamine (NAP) was classified as a group I carcinogen associated with bladder cancer. The daily exposure is mostly from cigarette and E-cigarette smoke. NAP can lead to testicular atrophy and interstitial tissue hyperplasia; however, the outcomes of NAP treatment on spermatogenesis and the associated mechanisms have not been reported. The study aimed to investigate the effect of NAP on spermatogenesis and sperm physiologic functions after being persistently exposed to NAP at 5, 20, and 40 mg/kg for 35 days. We found that sperm motility, progressive motility, sperm average path velocity, and straight-line velocity declined remarkably in the NAP (40 mg/kg) treated group, and the sperm deformation rate rose upon NAP administration. The testis immunity- and lipid metabolism-associated processes were enriched from RNA-sequence profiling. *Plvap*, *Ccr7*, *Foxn1*, *Trim29*, *Sirpb1c*, *Cfd*, and *Lpar4* involved in testis immunity and *Pnliprp1* that inhibit triglyceride and cholesterol absorption were confirmed to rise dramatically in the NAP-exposed group. The increased total cholesterol and CD68 levels were observed in the testis from the NAP-exposed group. *Gpx5*, serving as an antioxidant in sperm plasma, and *Semg1,* which contributes to sperm progressive motility, were both down-regulated. We concluded that the short-term exposure to NAP caused reproductive toxicity, primarily due to the inflammatory abnormality in the testis.

## 1. Introduction

2-naphthylamine (NAP), containing two fused aromatic rings, is referred to as a kind of polycyclic aromatic hydrocarbon (PAH) [1]. Occupational exposure to 2-naphthylamine (NAP) primarily takes place in the laboratories employing NAP for cancer research; the workers are exposed to NAP from fumes after pyrolysis and from the metabolic process of nitric polycyclic aromatic hydrocarbons in dye and rubber industrial production [2]. Notably, recent research found that cigarettes, including e-cigarette smoke, and the fumes from heating food oils, are the main sources of general population exposure [3]. NAP has been identified as one of the aromatic carcinogens belonging to group I carcinogens of urinary bladder carcinoma by the International Agency for Research on Cancer [4].

The absorption of NAP occurs through the skin, mucous membranes, inhalation, and gastrointestinal tract, and NAP in the body is mainly excreted in the urine after metabolism or in initial conditions and shows genotoxicity by forming DNA adducts in the bladder and liver [2]. People were subjected to dysuria, painful urination, and blood urine, as well as chronic contact dermatitis after exposure to NAP for the sensitization test [5]. The worker’s co-exposure to NAP and benzidine caused the relative quantity of CD4+ CD45RA+ T lymphocytes to decline substantially compared with that in the control group without occupational exposure to any hazardous chemicals [6]. The cohort studies indicated that the number of individuals suffering from cancers in various tissues, including bladder [7,8], stomach [9], lung [8], pancreas [9], kidney [10], prostate [9], testis [9], etc. were sharply increased after NAP exposure (production or use). In addition, testicular atrophy and interstitial tissue hyperplasia were observed in a small number of male rats showing pathological morphology after oral treatment of NAP five times a week lasting for one year, and interstitial cell tumors were observed in male rats’ testis [11]. In female rats, a high incidence of uterine polyps and endometrial hyperplasia was found after NAP treatment [11]. However, the evidence of NAP health risk to human reproduction was less evaluated, and the assays performed by animal models are also rare.

Benzo(a)pyrene [B(a)P], a typical PAH, is emitted mainly from cigarette smoking, vehicle traffic, carbonization, etc., and has a detrimental effect on male and female fertility by decreasing sperm production and oocyte maturation, respectively [1]. Considering the health risk of NAP not just from occupational exposure but also derived from cigarette smoke to the general population, we carried out the study to further characterize NAP reproductive toxicity to testis in a short-term administration. The objective is to investigate the effect of NAP on spermatogenesis employing a mouse model by intragastric treatment. Our data prove the worsening outcomes of NAP imposed on sperm motility and morphology, as well as the disorder of testicular immunity followed by NAP exposure in mouse testis.

## 2. Materials and Methods

### 2.1. Chemicals, Animals, and Study Design

The NAP (CAS: 91-59-8) was purchased from Adamas-beta (Shanghai, China). The lethal doses 50 (LD50) of NAP to rats was indicated by experimental studies at 727 mg/kg. The carcinogenicity of NAP was investigated through distinct exposure routes, including oral administration, subcutaneous injections, and intraperitoneal injections [2]. Thus, the intragastric administration of NAP is an effective approach for animal poisoning. The intragastric concentration of NAP was determined following previous studies carried out with mice via gavage [12,13]. The 6-week male ICR mice were purchased from the animal laboratory center, Nantong University, and housed in a 12 h:12 h cycle of light/dark in laboratory conditions, with feeding and drinking freely. After acclimatization for 3 days, 4 groups of 8 mice were randomized, and the intragastric administration was performed with NAP (dissolved in corn oil) or corn oil once daily for 35 running days. The mice were weighed every week, and the physiological state was under close evaluation. The mice were sacrificed 24 h after the last treatment, and the testis and epididymis were collected for further assays. All animal protocols and procedures were approved by the ethics committee of Nantong University (S20210224-039).

### 2.2. H&E Staining

Briefly, clearing the paraffin of the 5 μm paraffin sections in xylene and hydrating the samples, the sections were stained with hematoxylin for 5 min before blue turning in running tap water for 10 min. The slides were counterstained with eosin for 20 s, prior to placing them into xylene for another 10 min. The slides underwent microscopic examination (Zeiss, Jena, Germany) after adding coverslips.

### 2.3. Periodic Acid–Schiff Stain (PAS) Staining

The PAS staining was performed following the manufacturer’s instructions (Solarbio, Beijing, China). Briefly, the testis was oxidated by PAS oxidation in a dark place for 30 min, after the paraffin sections dewaxing and then stained by Schiff for 1 h. The nucleus was counterstained by hematoxylin. The tissue sections were examined under an optical microscope (Zeiss, Jena, Germany). The diameter of the seminiferous tubule and the thickness of germinal epithelium were acquired by ImageJ plugins (version 1.48).

### 2.4. Immunofluorescence

The 5 μm paraffin sections from mouse testis were used for immunofluorescence assay. The experimental procedures were based on our previous study [14]. The antibodies against DDX4 (1:100, ab13840, Abcam, Cambridge, MA, USA), PNA-FITC (10 μg/mL) (Thermo Fisher, Waltham, MA, USA), CD68 (1:200, 28058-1-AP, Proteintech, Wuhan, China), and Cy5 conjugated Goat Anti-rabbit IgG (H+L) (1:500, GB27303, Servicebio, Wuhan, China) were applied here. The images were acquired under a fluorescence microscope (Zeiss, Jena, Germany).

### 2.5. Sperm Motility Assessment by Computer-Aided Sperm Analysis (Casa)

The human tubal fluid (HTF) media was preheated in 37 °C, and the formula of HTF was as follows: NaCl (1.01 M), KCl (46.9 mM), MgSO_4_-7H_2_O (2 mM), CaCl_2_-2H_2_O (20.4 mM), glucose (55.5 mM), sodium lactate (3.56 mM), sodium pyruvate (3.32 mM), KH_2_PO_4_ (3.70 mM), and NaHCO_3_ (249 mM) (Sigma Aldrich, St. Louis, MO, USA), with the pH at 7.2–7.4. Briefly, the sperm was dissociated from the cauda epididymis together with the seminiferous duct prior to incubating with HTF for 10 min and 60 min at 37 °C. The physiological properties of capacitated sperm were performed using a sperm-counting chamber (80 μm, Songjing Tianlun, Nanning, China) with 15–20 μL dilution every time. Sperm motility parameters were measured by a computer-assisted sperm analysis (CASA) system (Hamilton Thorne CEROS, Beverly, MA, USA). At least 1500 sperm were collected at each time to analyze the motion parameters. The five motion parameters of sperm motility, progressive motility, straight-line velocity (VSL), curvilinear velocity (VCL), and average path velocity (VAP) were analyzed.

### 2.6. Acrosome Reaction

After incubation in HTF for 40 min, the capacitated sperm (100 µL) were treated with 1 μL ionomycin (Aladin, Shanghai, China) dissolved in HTF for another 40 min to induce acrosome reaction, with the sperm incubated in DMSO (0.014 μM in HTF) as a control (spontaneous acrosome reaction), and then the sperm was adhesived on slides before fixing with methanol for 30 s. The sperm was stained with PNA-FITC (10 μg/mL) (Thermo Fisher, Waltham, MA, USA) after rinsing 3 times with PBS and staying overnight in a dark place at 4 °C. The sperm were counterstained with DAPI (Beyotime, Shanghai, China) for 3 min after washing by PBST for 3 cycles. The fluorescence imaging was acquired by Axio Imager M2 microscopy (Zeiss, Jena, Germany). The total sperm stained with PNA-FITC from each mouse was counted in 300 views, and the acrosome reaction rate was calculated by the number of sperm undergoing acrosomal reactions/total views (300), with three mice in each group. The acrosome reaction was determined according to the degree of acrosome disappearance/destruction.

### 2.7. Malformation Detection

The high-saline solution (HS) containing NaCl (135 mM), KCl (5 mM), MgSO_4_·7H_2_O (1 mM), CaCl_2_·2H_2_O (2 mM), HEPES (20 mM), glucose (5 mM), 10 mM lactic acid, 1 mM Na pyruvate (Sigma, St. Louis, MO, USA) was prepared, with the pH at 7.4. The clipped cauda epididymis into HS (1 mL) was incubated at 37 °C for 10 min, and then the incubation medium (900 μL) was centrifuged at 300 rcf for 6 min. Discarding the supernatant and remixing the pellet, 30 μL sperm was coated in an adhesive slide before observing sperm morphology by Cap Studio (iMG, Suzhou, China). The deformity rate was calculated by evaluating the deformed sperm in a total of 200 sperm from each mouse, with four mice in each group.

### 2.8. RNA Extraction, RNA-Sequencing, and qRT-PCR

Briefly, Trizol reagent (Sigma, MO, USA) was used for testicular RNA extraction based on the manufacturer’s instructions. RNA integrity was validated by agarose gel electrophoresis (1%), before examining the concentration and purity by NanoDrop One Microvolume UV-Vis Spectrophotometer (Thermo, MA, USA).

RNA library was constructed by NEB Next^®^ Ultra™ RNA Library Prep Kit for Illumina^®^ (NEB, Ipswich, MA, USA) based on the manufacturer’s instruction. Briefly, RNA sequencing (RNA-seq) was carried out by Genewiz Ltd. Co. (Suzhou, China). The genetic difference analysis was performed by DESeq R package (1.10.1), and the significant difference would be considered when *p* < 0.05. Gene ontology (GO) annotation was analyzed by GOseq R packages, and the Kyoto Encyclopedia of Genes and Genomes (KEGG) orthology enrichment was carried out by the KO-Based Annotation System 2.0 (KOBAS) software.

The reverse transcription and qRT-PCR assays were carried out by the kits of HiScript III RT Supermix for qPCR and ChamQ Universal SYBR qPCR Master Mix (Vazyme, Nanjing, China), respectively. β-actin was used as a reference to standardize the cycle threshold (CT) values of desired genes after RNA quantification by the Roche Light Cycler sequence system (Roche Diagnostics, Basel, Switzerland). mRNA fold variations were determined by the ΔΔCT approach. All gene primers are shown in Table 1.

### 2.9. The Detection of Total Cholesterol in Testis

The samples in BeyoLysis™ Buffer A (Beyotime, Nanjing, China) at equivalent weight were homogenized at 4 °C, then centrifuged at 12,000× *g* at 4 °C for 5 min and the supernatant for the detection. Cholesterol detection assays were carried out using Amplex Red Cholesterol and Cholesterol Ester test kit (S0211S, Beyotime, China) based on the manufacturer’s instructions. The concentration was determined by absorbance at 570 nm.

### 2.10. Statistical Analysis

The numerical results were shown as the mean ± standard error of mean (SEM). An unpaired two-tailed *t*-test was applied to analyze the difference between the two groups, and when the multiple comparisons were being carried out, the one-way ANOVA with the Tukey test was employed. *p*-values < 0.05 were regarded as significant differences.

## 3. Results

### 3.1. The Histological Patterns of the Testis and Epididymis Were Not Remarkably Injured after NAP Treatment

**Figure 1:**
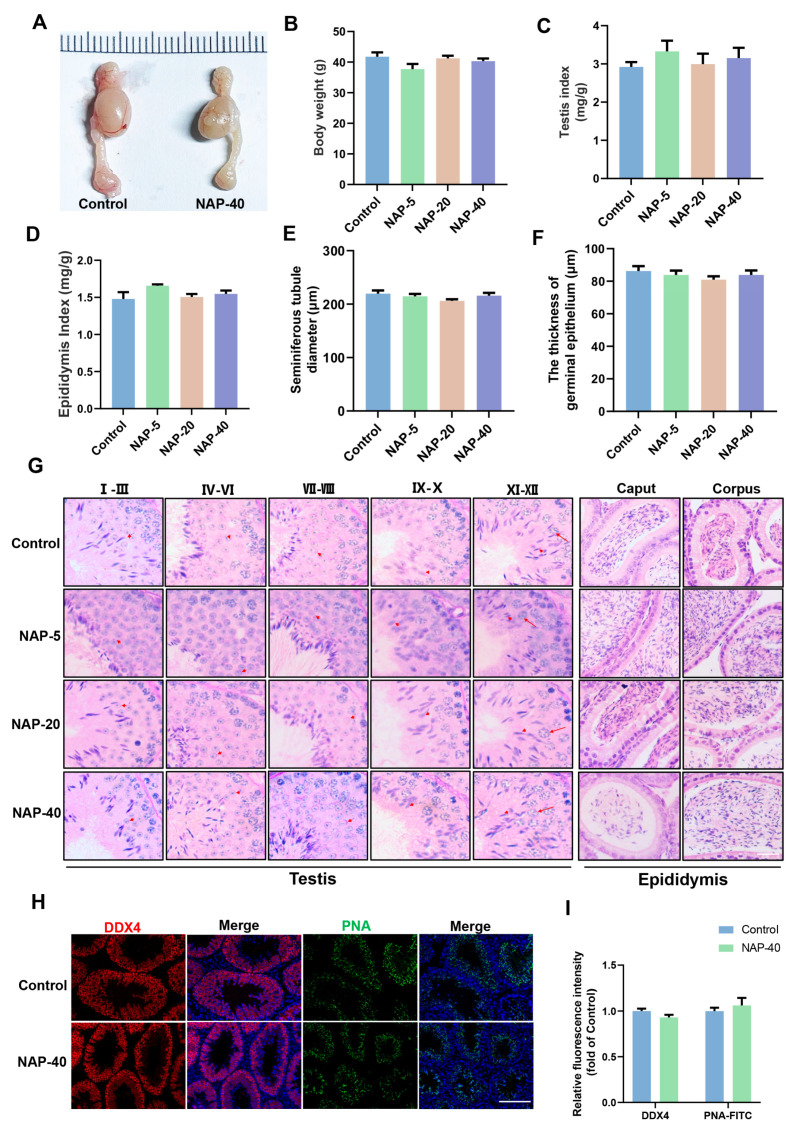
The effect of 2-naphthylamine (NAP) on anatomical morphology, organ index, histological morphology of the testis and epididymis, and DDX4, PNA expression in testis. (**A**) The anatomical morphology of testis and epididymis. (**B**) The body weight of mice after exposure to NAP. (**C**,**D**) The organ index of the testis and epididymis showed no variance among groups. n = 6, 4, 4, 6. (**E**,**F**) The diameter of the seminiferous tubule and the thickness of the germinal epithelium (n = 10). (**G**) NAP treatment does not influence the staging of seminiferous tubules and the histological patterns of caput and corpus epididymis following NAP exposure. The short arrows indicate spermatids stained with PAS, while the long arrows show the spermatocytes. n = 3, bar = 20 μm. (**H**) The cellular location of DDX4 and PNA in testis was determined by immunofluorescence. n = 3, bar = 50 μm. (**I**) Quantitative graphs were shown as the expression of DDX4 and PNA in the control and NAP-40 groups, calculated by ImageJ. n = 15.

After NAP exposure for 35 days, the development of the reproductive system was evaluated. Figure 1A indicated that the anatomical testis and epididymis were comparable between the control and NAP group (40 mg/kg, NAP-40). The body weight (Figure 1B), testis, and epididymis index were similar among groups (5 mg/kg, NAP-5; 20 mg/kg, NAP-20, NAP-40) (Figure 1C,D).

Figure 1G indicates the testicular morphology and acrosomal patterns performed by PAS staining. There are 12 stages in total of the seminiferous epithelial cycle during spermatogenesis, based on the location of spermatogenic cells (spermatogonia, spermatocyte, and haloid sperm) in the seminiferous tubule and the acrosome pattern of round sperm [15,16]. Additionally, spermiogenesis in mice can be divided into 16 steps based on acrosome biogenesis [17]. The integrated seminiferous tubules’ morphology appeared in NAP-treated groups and indicated that spermatogenesis was in normal behaviors after NAP administration by identifying the acrosome morphology in the distinct spermatogenesis stages at I–III, IV–VI, VII–VIII, IX–X, and XI–XII (Figure 1G). The diameters of the seminiferous tubule and the thickness of germinal epithelium have no significant difference (Figure 1E,F). The histomorphology of caput and corpus epididymis was present, and the epididymal epithelium is undamaged, with spermatozoa distributing in distinct segments of the epididymis (Figure 1G). The expression patterns of DDX4 (germ cell marker) and PNA (spermatid marker) were similar between the control and NAP-40 groups (Figure 1H,I).

### 3.2. The Sperm Motility and Morphology Deteriorated following NAP Exposure

**Figure 2:**
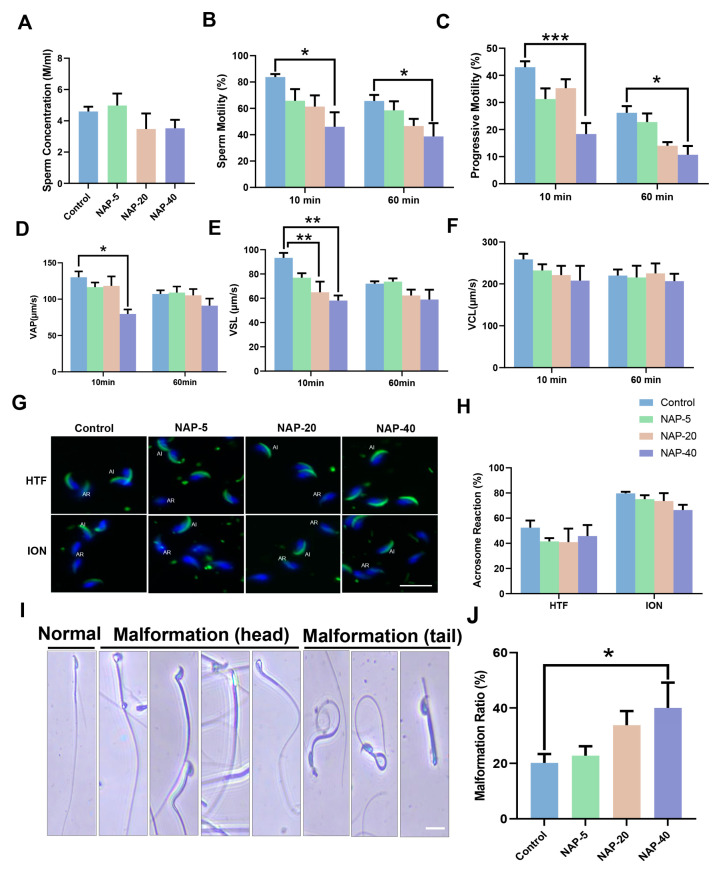
The effect of 2-naphthylamine (NAP) on sperm concentration, physiological functions, and malformation. (**A**) Sperm concentration was not reduced markedly in the NAP-exposed group. n = 5. (**B**,**C**) The sperm motility (n = 5, 4, 5, 3) and progressive motility (n = 5, 4, 4, 3) rate dropped significantly in the NAP-40 group after incubation with HTF for 10 min or 60 min. (**D**,**E**) VAP (n = 5, 4, 5, 3) and VSL (n = 4, 4, 3, 3) fell significantly following NAP treatment at 40 mg/kg and at 20, 40 mg/kg, respectively. (**F**) No notable effect was found in VCL upon NAP exposure. n = 5, 4, 4, 5. (**G**) The mouse sperm acrosome reaction was detected by PNA-FITC staining, the acrosome integrity (AI) indicates uncapacitated sperm, and acrosome reaction (AR) suggested sperm in acrosome-reacted condition. Scale bar = 20 μm. (**H**) No significant changes were found in acrosome reaction rate either in spontaneous condition or after ionomycin stimulation. (**I**) The sperm malformation ratio rose sharply in the NAP-40 treated group. * *p* < 0.05, ** *p* < 0.01, *** *p* < 0.001. n = 5, 4, 4, 3. (**J**) The multiple types of sperm malformation observed in NAP exposure groups, including sperm head bending, curled or fractured tails, etc. Scale bar = 20 μm.

Next, the sperm’s physiological characteristics were evaluated, including sperm concentration, motility, acrosome reaction rate, and malformation. The NAP exposure did not create defects in sperm concentration despite a downward trend found in the NAP-20 and NAP-40 groups (Figure 2A). The sperm motility and progressive motility rate declined substantially in the NAP-40 group, both 10 min and 60 min after incubation with HTF (Figure 2B,C). The VAP decreased at 10 min in the NAP-40 group (Figure 2D) and VSL went down in the NAP-20 and NAP-40 groups sharply (Figure 2E).

As shown in Figure 2G, the spontaneous acrosome reaction (AR) (HTF group) and induced AR (ionomycin (ION)-stimulated) were observed upon NAP treatment; there is no variation in the acrosome reaction rate in treatment groups compared with control subjects either in the HTF incubated group or after ION exposure (Figure 2H). The rate of sperm malformation, including head and tail formation, rose sharply in the NAP-40 group (Figure 2J). Figure 2I indicated various types of sperm malformations such as sperm head bending, curled tails, fractured tails, etc.

### 3.3. NAP Disturbed Gene Expression Profiles in the Testis

**Figure 3:**
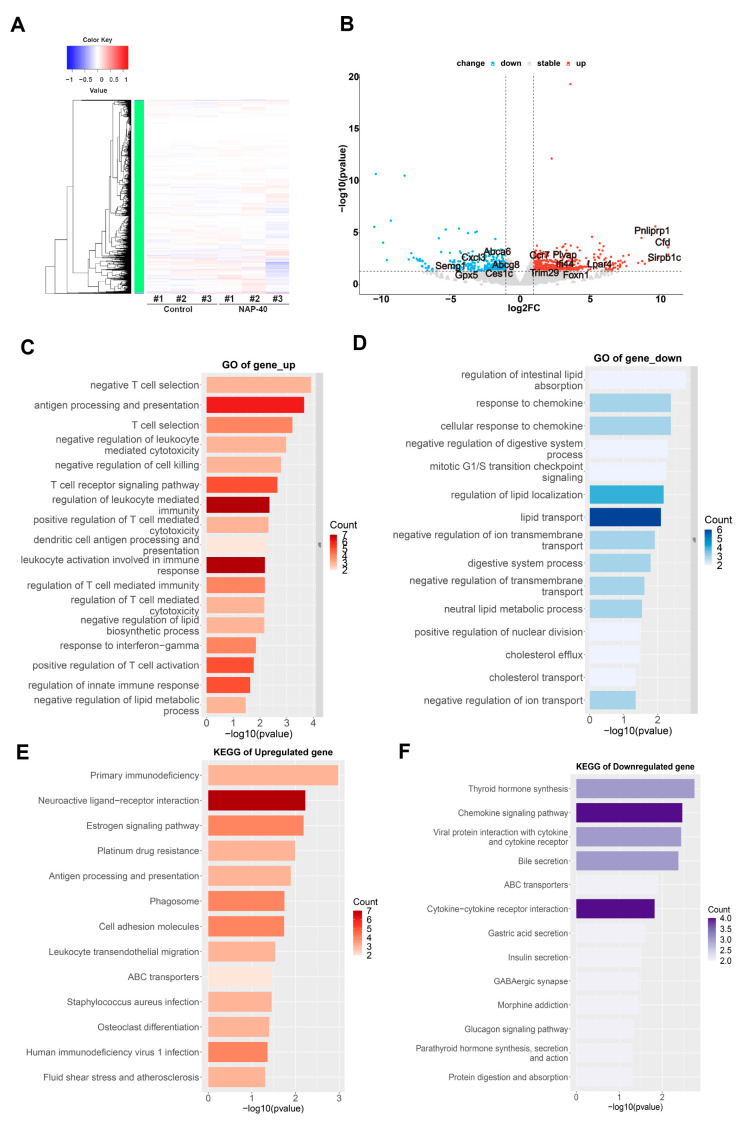
The mRNA profiling analysis by gene ontology (GO) annotation and Kyoto Encyclopedia of Genes and Genomes (KEGG) mapping enrichment between the control and NAP-40-treated group. (**A**) The matrix heatmap of differentially expressed genes. (**B**) The up-regulated (421) and down-regulated genes (286) were shown in the volcano plot. (**C**,**D**) GO terms were enriched in differential genes. (**E**,**F**) The metabolic pathways were annotated from the KEGG database. n = 3.

To explore the underlying mechanism of the reproductive toxicity upon NAP administration, RNA-seq was carried out to evaluate the transcriptional profile variation between the NAP-40 exposed and control group. Figure 3A shows the matrix heatmap of differential genes, with a total of 421 up-regulated genes and 286 down-regulated genes in the testis in response to NAP stimulation that was shown by the volcano plot (Figure 3B). Up-regulated gene analysis by GO annotation was enriched in antigen processing and presentation, negative regulation of leukocyte, T cell receptor signaling pathway, regulation of leukocyte mediated immunity, dendritic cell antigen processing and presentation, response to interferon-gamma, regulation of innate immune response, etc. (Figure 3C). Additionally, the highly enriched lipid metabolism-related pathways were screened among down-regulated genes, including the regulation of lipid localization, lipid transport, neutral lipid metabolic process, cholesterol efflux, etc. Notably, the cellular response to the chemokine process was repressed in the NAP-40 group (Figure 3D). The pathway mapping based on KEGG revealed primary immunodeficiency, antigen processing and presentation, leukocyte transendothelial migration, etc., were elevated (Figure 3E), and the chemokine signaling pathway was down-regulated (Figure 3F).

### 3.4. Testicular Immunity, Lipid Metabolism, and Sperm Motility Regulation Were Disturbed after NAP Exposure

**Figure 4:**
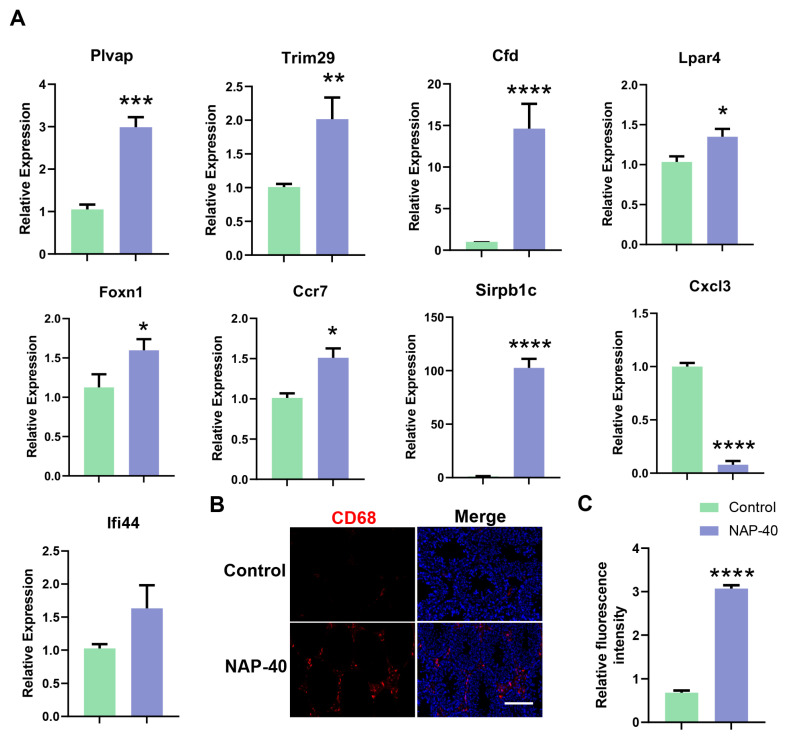
2-naphthylamine (NAP) treatment caused testicular immunity disorders. (**A**) The differential genes were quantified by qRT-PCR, including *Plvap*, *Trim29*, *Cfd*, *Lpar4*, *Foxn1*, *Ccr7*, *Sirpb1c*, *Ifi44*, and *Cxcl3*. * *p* < 0.05, ** *p* < 0.01, *** *p* < 0.001, **** *p* < 0.0001. n = 9. (**B**) CD68+ macrophage accumulation in the testis was positively related to NAP stimulation. Bar = 50 μm. (**C**) The comparison of CD68 expression was quantified by Cy5 fluorescence. n = 8.

**Figure 5:**
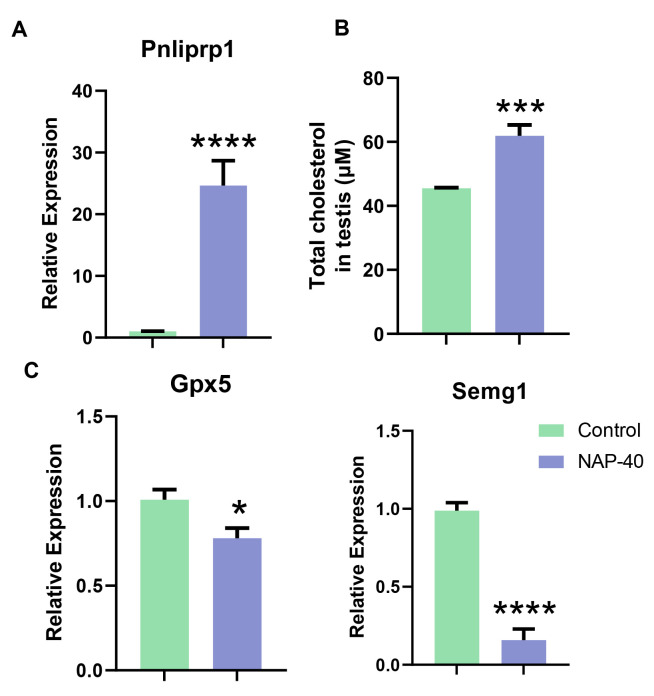
The dysregulation of lipid metabolism, as well as sperm motility in 2-naphthylamine (NAP)-treated mice. (**A**,**C**) The mRNA levels of *Pnliprp1*, *Gpx5,* and *Semg1* were compared between two groups. n = 9. (**B**) NAP exposure induced total cholesterol accumulation in mouse testis. n = 6. * *p* < 0.05, *** *p* < 0.001, **** *p* < 0.0001.

The differential genes being relevant to immunity, lipid digestion, and sperm motility were screened based on RNA expression profile analysis. Our findings showed the levels of C-C Motif Chemokine Receptor 7 (*Ccr7*), plasmalemma vesicle-associated protein (*Plvap*), tripartite interaction motif 29 (*Trim29*), Forkhead Box N1 (*Foxn1*), and signal regulatory protein beta 1 C (*Sirpb1c*) was increased dramatically in testis after NAP exposure. C-X-C Motif Chemokine Ligand 3 (*Cxcl3*) was shown to have dropped significantly in the NAP-40 treated group. The complement factor D (*Cfd*) and lysophosphatidic acid receptor 4 (*Lpar4*) increased substantially upon NAP-40 stimulation (Figure 4A). CD68, which is regarded as a general biomarker of macrophages and other mononuclear phagocytes [18], was highly expressed in mouse testis after NAP administration (Figure 4B,C). Further, we identified pancreatic lipase-related protein 1 (*Pnliprp1)* depressing dietary triglyceride, cholesterol digestion went up remarkably (Figure 5A), and testicular total cholesterol was found to increase (Figure 5B). Glutathione peroxidase 5 (*Gpx5*) and Semenogelin 1 (*Semg1*), which were involved in sperm motility, decreased sharply in the treatment group (Figure 5C).

## 4. Discussion

The available data indicated that B(a)P caused sperm count, progressive motility, and morphological abnormalities increased significantly versus control counterparts [1]. Similarly, our study showed that reproductive toxicity was caused in male mice after NAP poisoning, indicated by impaired sperm motility and higher sperm malformation. The declining sperm concentration was observed in the NAP-20 and NAP-40 groups, although less dramatically, with normal testicular histologic characteristics in the NAP-exposed group, indicating the toxic behavior could be dose- and time-dependent. Furthermore, the blood–testis barrier (BTB) in the seminiferous tubule contributes to spermatogenesis by separating spermatogonia and preleptotene spermatocytes in the basal compartment and other advanced germ cells in the adluminal compartment. BTB protects spermatogenic cells from lymphatic and circulatory systems, providing an immunosuppressive microenvironment for spermatozoa production [19]. Moreover, BTB-related gene profiles were normal in the NAP-40 exposed group, and it protects the testis from adverse effects after NAP treatment to some extent. Being different from accumulated evidence that the mutagenic and carcinogenic effects were caused in humans or experimental animal models [2], the differential genes associated with genotoxicity in testis followed NAP exposure were not enriched, primarily due to the shortened experiment period. A range of gene expression profiles related to testis immunity, lipid metabolism, and sperm physiological functions were aberrant, including *Plvap*, *Trim29*, *Cfd*, *Lpar4*, *Ccr7*, *Foxn1*, *Sirpb1c*, *Cxcl3*, *Pnliprp1*, *Gpx5,* and *Semg1*.

PLVAP is found in the cell surface, regulates cell cellular extravasation positively, and is involved in the TNFA signaling cascade [20,21]. TRIM29 exerts a vital role in macrophage activation in the respiratory tract in response to pathogen infection [22]. LPAR4 was involved in both innate and adaptive immunity via mediating monocytic differentiation [23,24]. CFD was involved in the rate-limiting step in the complement activation pathway [25], and the interaction of complement with T cells or macrophages is crucial in the regulation of immune response [26,27]. Consistently, the number of CD68+ macrophages increased following NAP treatment in mouse testis. Macrophages are the predominantly leukocyte subpopulation in the testis, and they provide efficient protection against invading pathogen infection for the developing germ cells, contributing to maintaining testis homeostasis and testicular development [28]. However, persistent infection and inflammation are associated with male reproductive disorders, including leukocytic infiltration, germ cell loss [29], sperm motility defect [30], and sperm malformation [31]. Our data showed that NAP treatment caused testicular macrophages to increase, further resulting in impaired sperm functions.

*Ccr7* belongs to the G protein-coupled receptor family localized in a variety of lymphoid tissues and mainly regulates T cell and B cell activation, dendritic cell maturation, T cell migration to inflamed tissues, and T cell homeostasis [32,33]. *Foxn1* promoted T-cell development in the early embryos [34], and its mutation is closely linked to T-cell immunodeficiency [35]. *Sirpb1c* plays a role in phagocytosis and T-cell activation [36]. Cytotoxic T-cells, a subset of T lymphocyte cells (CD8+ T cell), specifically secretes a variety of cytokines during the adaptive immune process, killing viruses, tumor cells, and other antigenic substances [37]. Sung et al. found that the number of CD4+CD45RA+ T cells in blood was significantly suppressed upon NAP treatment [6]. CD4+CD45RA+ T cells are indicated to help immunoglobulin synthesis from B cell through pokeweed mitogen (PWM)-induced pathway to a very small extent, while it induced CD8+ T lymphocytes in a suppressive state [38], indirectly proving CD8+ T cells were activated after NAP exposure. The current study indicated the genes mediating T lymphocyte’s physiological function were up-regulated in the testis, and this was similar to the previous surveys. Notably, we identified the cellular response to the chemokine process and chemokine signaling pathway were downregulated in the NAP-treated group. *Cxcl3*, serving a function in inflammation and promoting neutrophil chemotaxis was found to be dropped significantly after the NAP-40 treated group. The decreased *Cxcl3* indicated the chemotactic movement of neutrophils can be weakened, resulting in the testis susceptibility to infection.

The up-regulated genes were enriched in the negative regulation of the lipid metabolic process, and lipid localization, digestive system process, neutral lipid metabolic process, etc., were down-regulated, suggesting the lipid metabolism abnormality within the testis followed NAP stimulation. PNLIPRP1 suppressed the absorption of triglyceride and cholesterol when rising abnormally [39,40]. Endogenous and dietary lipid has dual functions in pro- and anti-inflammatory processes by promoting both M1 (pro-inflammatory type) and M2 macrophage (anti-inflammatory type) differentiation, and dysregulated lipid metabolism leads to the dysfunction of macrophage [41]. The lipid metabolic disturbance caused by NAP treatment may further damage sperm physiological properties by stimulating macrophage activation. Additionally, GPX5, which protects sperm membranes from peroxide damage by catalyzing the reduction of peroxide, decreased markedly and indicated a reduction in oxidation resistance. The lipid peroxidation accumulation and lower peroxide scavenging activity are correlated closely with impaired sperm motility [42,43] and midpiece abnormalities [42,44]. *Sem*g1-encoded protein is predominantly found in semen, and it contributes to sperm progressive motility activation by helping the synthesis of the gel matrix, which encases the secretions from the accessory gland and ejaculated spermatozoa [45,46]. The inflammatory response and oxidative stress were indicated in unilateral varicocele with symptoms of reduced sperm count and aberrant morphology [47,48], and SEMG1 was at a lower level and identified as a marker in unilateral varicocele patients [47], suggesting a negative correlation between SEMG1 level and inflammation, oxidation. Our results proved that *Semg1* was inhibited under the administration of NAP, further resulting in deteriorating sperm quality.

## 5. Conclusions

In summary, we determined that short-term exposure to NAP led to deteriorated sperm parameters, expanding the toxicological references of PAHs to male reproduction and promoting us to raise the prevention awareness of environmental NAP exposure. Apart from occupational exposure, the general population is more likely to be exposed to NAP through cigarette smoking in a short period; thus, identifying and preventing the reproductive toxicity of NAP to individuals falling within the “at-risk” group has positive implications for reducing potential health risks.

## Figures and Tables

**Table 1:** The sequences of gene primers.

Gene	The Sequence of Primers	Gene	The Sequence of Primers
*Ifi44*	F: AACTGACTGCTCGCAATAATGT R: GTAACACAGCAATGCCTCTTGT	*Cfd*	F: GCAAGTGAACGGCACACAC R: GAGTCGTCATCCGTCACTCC
*Trim29*	F: AGAATGGCACTAAAGCAGACAG R: AAATAGGCCACTCTTCCCCTC	*Pnliprp1*	F: CTTCTCCCTTGGAGCCCTGA R: GCCATGGATGATGAACCGAG
*Foxn1*	F: ATGGTGTCGCTACTCCCTCC R: AGGCACAAACGACGAGCAG	*Lpar4*	F: AGTGCCTCCCTGTTTGTCTTC R: GCCAGTGGCGATTAAAGTTGTAA
*Gpx5*	F: TCTAGCCAGCTATGTGCAGAC R: TCCTTCCCATTAAGAGACAGAGC	*Cxcl3*	F: CCCTACCAAGGGTTGATTTTGA R: GGCTATGACTTCTGTCTGGGTG
*Ccr7*	F: GTGGTGGCTCTCCTTGTCAT R: CTTGAAGCACACCGACTCGT	*Semg1*	F: TCTGTCTTCGTCCTTTCTCTGC R: GATCCCGAAACTGAGACGGC
*Plvap*	F: CATCGCCGCTATCATCCTGA R: AGCTTCGCAGGTCTTGTTGA	*β-actin*	F: GATCTTCATTGTGCTGGGTG R: GGGAAATCGTGCGTGACATT
*Sirpb1c*	F: CCATCAGAGCCTGACATGGA R: CTGCAGGACCGGAGACCATA		

## Data Availability

Pengyuan Dai (2024), “The male reproductive toxicity caused by 2-naphthylamine was related to testicular immunity disorders”, Mendeley Data, V1, doi: 10.17632/hzmh8wzzx5.1.
